# Segmentation of cellular ultrastructures on sparsely labeled 3D electron microscopy images using deep learning

**DOI:** 10.3389/fbinf.2023.1308708

**Published:** 2023-12-15

**Authors:** Archana Machireddy, Guillaume Thibault, Kevin G. Loftis, Kevin Stoltz, Cecilia E. Bueno, Hannah R. Smith, Jessica L. Riesterer, Joe W. Gray, Xubo Song

**Affiliations:** ^1^ Program of Computer Science and Electrical Engineering, Oregon Health and Science University, Portland, OR, United States; ^2^ Department of Biomedical Engineering, Oregon Health and Science University, Portland, OR, United States; ^3^ Knight Cancer Institute, Oregon Health and Science University, Portland, OR, United States; ^4^ Department of Medical Informatics and Clinical Epidemiology, Oregon Health and Science University, Portland, OR, United States

**Keywords:** electron microscopy, segmentation, deep neural network, cell boundary, subcellular ultrastructure

## Abstract

Focused ion beam-scanning electron microscopy (FIB-SEM) images can provide a detailed view of the cellular ultrastructure of tumor cells. A deeper understanding of their organization and interactions can shed light on cancer mechanisms and progression. However, the bottleneck in the analysis is the delineation of the cellular structures to enable quantitative measurements and analysis. We mitigated this limitation using deep learning to segment cells and subcellular ultrastructure in 3D FIB-SEM images of tumor biopsies obtained from patients with metastatic breast and pancreatic cancers. The ultrastructures, such as nuclei, nucleoli, mitochondria, endosomes, and lysosomes, are relatively better defined than their surroundings and can be segmented with high accuracy using a neural network trained with sparse manual labels. Cell segmentation, on the other hand, is much more challenging due to the lack of clear boundaries separating cells in the tissue. We adopted a multi-pronged approach combining detection, boundary propagation, and tracking for cell segmentation. Specifically, a neural network was employed to detect the intracellular space; optical flow was used to propagate cell boundaries across the z-stack from the nearest ground truth image in order to facilitate the separation of individual cells; finally, the filopodium-like protrusions were tracked to the main cells by calculating the intersection over union measure for all regions detected in consecutive images along z-stack and connecting regions with maximum overlap. The proposed cell segmentation methodology resulted in an average Dice score of 0.93. For nuclei, nucleoli, and mitochondria, the segmentation achieved Dice scores of 0.99, 0.98, and 0.86, respectively. The segmentation of FIB-SEM images will enable interpretative rendering and provide quantitative image features to be associated with relevant clinical variables.

## 1 Introduction

Recent advances in tumor biology have shown that the plethora of interactions between tumor cells and their surrounding environment can significantly influence the behavior of cancer and its response to treatment (Hirata and Sahai, 2017; Tanaka and Kano, 2018; Galli et al., 2020). A deeper understanding of the underlying cellular mechanisms will shed light on how cancer evolves and develops resistance to therapy (Thakkar et al., 2020). The understanding of these dynamic interactions can be used to develop novel approaches to disrupt key inter- and intracellular interactions and facilitate the design and development of efficient therapeutic strategies to fight cancer (Baghban et al., 2020).

Electron microscopy (EM) provides nanometer resolution views of intra- and intercellular interactions that are not apparent in images generated using light microscopy (Johnson et al., 2020). This complete picture of spatial relationships can reveal potential therapeutic targets that can be related back to the macroscale heterogeneity and microenvironment of the tissue. Focused ion beam-scanning electron microscopy (FIB-SEM) is especially informative, generating stacks of 2D SEM images that provide 3D information about subcellular features in large tissue volumes (Giannuzzi, 2004). FIB-SEM imaging proceeds via serial steps of SEM imaging of a sample surface and FIB removal of a uniform thin layer of the tissue with a depth comparable to the spatial resolution in the x-y plane, thereby revealing a new surface to be imaged. This process is fully automated and ensures that imaged data are equidimensional in all three axes, which significantly improves the accuracy of feature recognition within the dataset.

**FIGURE 1:**
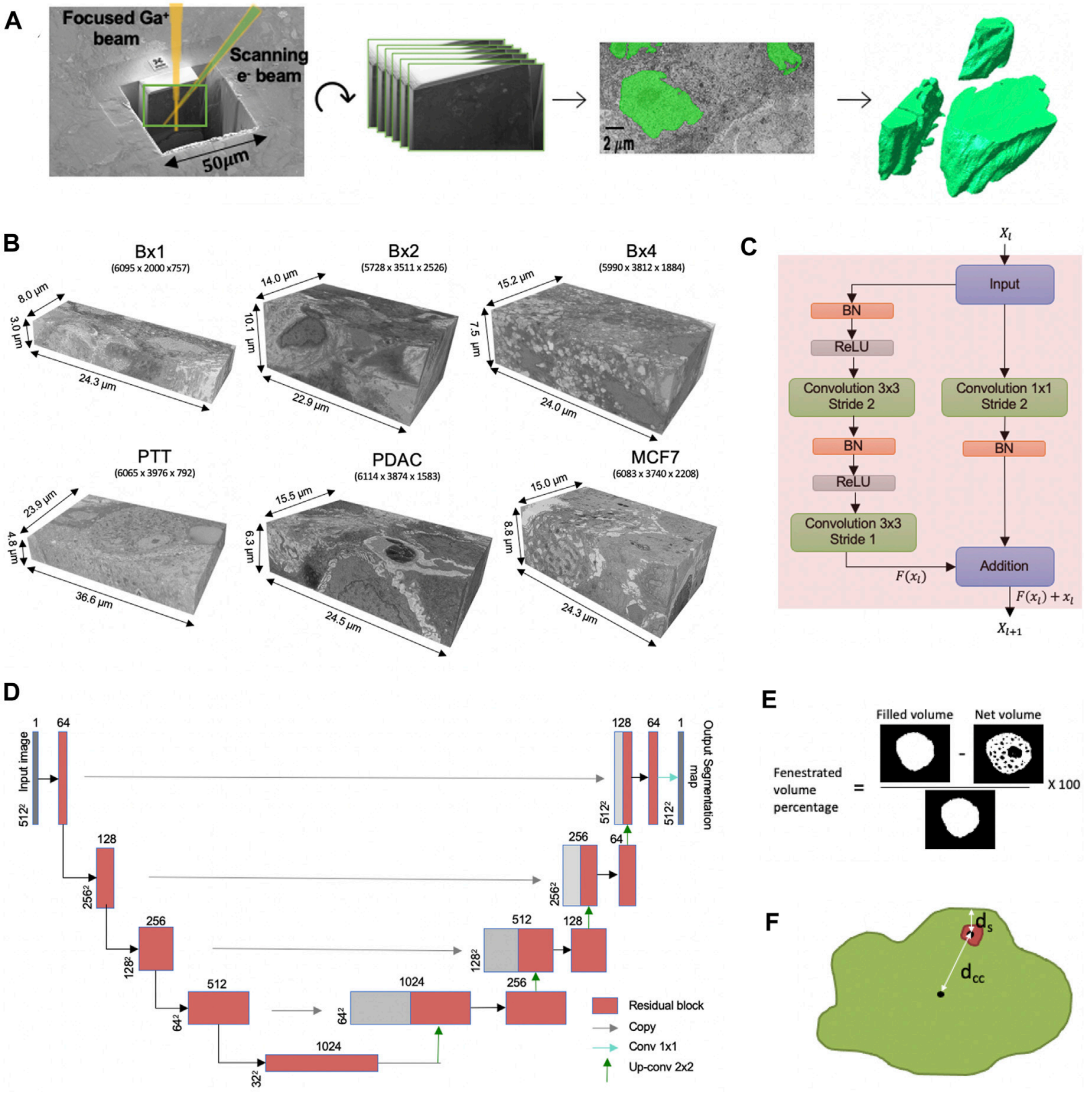
**(A)** FIB-SEM-to-volume rendering workflow. The FIB source sequentially slices a few nanometers from the sample to expose a fresh surface for subsequent imaging using the electron beam. An image stack is acquired, and after image alignment and cropping, a small subset of the stack of images was segmented manually to generate a training set for the deep learning model. Once trained, the deep learning model is used to predict segmentation masks for the rest of the images in the stack. These predictions are used to create volume renderings for the examination of 3D ultrastructural properties. **(B)** Six FIB-SEM datasets and their sizes. The 3D FIB-SEM volumes collected from the biopsy samples Bx1, Bx2, and Bx4 acquired from a patient with metastatic breast ductal carcinoma, two biopsy samples (PTT and PDAC) acquired from two patients with pancreatic ductal adenocarcinoma, and a microspheroid prepared using a breast cancer cell line (MCF7). **(C)** Residual block used in ResUNet. BN stands for batch normalization, and ReLU stands for rectified linear unit. *X*_*l*_ and *X*_*l*+1_ are the input and output features for the residual layer *l*, respectively, and *F* represents the residual function. **(D)** ResUNet architecture. Input size is written on the side of each box. The number of feature maps in each residual layer is written on top of each box. **(E)** Illustration of the net volume and filled volume used in the fenestrated volume percentage measure (3D volume is represented as a 2D image) and **(F)** illustration of distances *d*_*cc*_ and *d*_*s*_ used in proximity of the nucleolus to the nuclear membrane measure.

While 3D FIB-SEM images are being generated with an ever increasing rate in ongoing research and clinical programs, the rate-limiting step in their analysis is the delineation of the cellular structures to enable rendering of the images into interpretable forms. Currently, this is done by experts manually annotating images and, while effective, is extremely time-consuming, tedious, and dependent on the skill of the expert. The development of rapid, robust, and automated machine learning methods to segment ultrastructural features is acutely needed for the widespread use of EM in large-scale studies (Perez et al., 2014). The ultrastructure refers to the structure of the cell and its organelles that are visible only with high magnification and highest obtainable resolution. The workflow from FIB-SEM imaging to volume rendering in shown in Figure 1A and is commonly referred to as volume electron microscopy (vEM).

## 2 Related work

Early research on automated EM segmentation focused on neuronal ultrastructure segmentation of brain tissue. Two-dimensional U-Net and its variants using residual connection, such as FusionNet (Quan et al., 2016), fully connected networks (FCNs) with skip connections (Drozdzal et al., 2016), and M2FCN (Shen et al., 2017), have been proposed for neuronal membrane segmentation in EM images to yield large neurite superpixels combined into 3D neuronal objects using iterative region agglomeration algorithms. Two-dimensional convolutional neural networks (CNNs) have the advantage of requiring only 2D ground truth and having a computationally inexpensive training process (Ishii et al., 2020). Recent automated neurite reconstruction methods use 3D CNNs to yield highly accurate 3D boundary probability maps requiring a simple watershed algorithm for final segmentation (Zeng et al., 2017; Linsley et al., 2018).

More recently, efforts have been made toward segmentation of organelles in EM images of non-neuronal tissues. Three-dimensional U-Net followed by the mutex watershed algorithm was used to segment nuclei, and the lifted multicut-based approach was used for cell segmentation to characterize the morphology of cells in an EM volume of a complete *Platynereis* worm (Vergara et al., 2021). Insulin secretory granules and Golgi apparatus segmented using 3D and 2D U-Net, respectively, helped in generating a comprehensive spatial map of organelle interactions in mouse *β* cells and facilitated our understanding of the supportive role played by secretory granules in insulin secretion (Müller et al., 2021). Three-dimensional U-Net was used to segment 35 organelles from EM images of HeLa, Jurkat, macrophage, and SUM159 cells (Heinrich et al., 2021). Subcellular structures of liver tissue in mice segmented using 3D U-Net helped demonstrate substantial alterations in the hepatic endoplasmic reticulum of lean and obese mice (Parlakgül et al., 2022). Although the 3D CNNs exhibit enhanced performance, they necessitate a greater number of labeled images for training.

The segmentation of individual cells from EM images is an essential step to perform quantitative cellular analysis. However, the ultrastructure of tumor cells and the tumor microenvironment is different from that of the widely studied neuronal and other normal cells (Coman and Anderson, 1955; Zink et al., 2004; Baba and Câtoi, 2007). The segmentation methodologies designed for the neuronal cells use cell boundaries as the strongest cue to delineate a cell (Shen et al., 2017; Linsley et al., 2018; Müller et al., 2021; Vergara et al., 2021). Due to the lack of clear boundaries separating cells in cancer tissues, segmentation methodologies designed for neuronal cells cannot be directly applied for cancer cell segmentation. The convoluted and intertwined nature of cancer cells and the presence of filopodium-like protrusions make it even more challenging (Johnson et al., 2020). Recently, cells were segmented in EM images of hepatoblastoma patient-derived xenograft tissue. The method involved labeling cells in every 10th image and using optical flow to propagate the contour from these labeled images onto the neighboring images (de Senneville et al., 2021). Propagation-based methods can only propagate the contour of the cells labeled in the ground truth images. However, the filopodium-like protrusions present in cancer cells appear as island-like blobs detached from the main cell (Supplementary Video S1), and a propagation-based method cannot track such islands if they are not present in the ground truth image.

## 3 Methods

### 3.1 3D focused ion beam-scanning electron microscopy dataset collection

Under an Institutional Review Board (IRB)-approved observational study, three tissue biopsy samples (Bx1, Bx2, and Bx4) were acquired over three time points of cancer treatment from a patient with metastatic ER + breast ductal carcinoma, and two biopsy samples (PTT and PDAC) were acquired from two patients with pancreatic ductal adenocarcinoma at Oregon Health and Science University, Portland. The last sample is a microspheroid prepared using a breast cancer cell line (MCF7). Extensive additional information about the three biopsies from the breast cancer patient is available (Johnson et al., 2020; Johnson et al., 2022). The samples were preserved in Karnovsky’s fixative (2.0% PFA and 2.5% gluteraldehyde), post-fixed using an OsO_4_–TCH–OsO_4_ staining protocol (Riesterer et al., 2020; Stempinski et al., 2023), and embedded in EPON resin. Post-fixation staining binds heavy metals to lipid-rich membranes to provide contrast in EM imaging. Conductive coating with 8-nm-thick carbon was necessary to achieve high-resolution, charge-free, high-contrast, and low-noise images. A FEI Helios NanoLab 660 DualBeam™ microscope was used to collect high-resolution 3D volumes of the resin-embedded blocks. Targeted volumes were collected using a Ga^+^ FIB source to sequentially slice a few nanometers from the sample to expose a fresh surface for subsequent imaging. The slicing/imaging cycle was automated using FEI Auto Slice and View™ software extended package, while the in-column detector (ICD) was used for image collection during 3D data acquisition. Metastatic breast cancer, primary pancreatic tissues, and the microspheroid were imaged with an isotropic resolution of 4, 6, and 6 nm, respectively.

### 3.2 Image preprocessing and ground truth generation

After data acquisition, images within the stack are translationally aligned in the x-y plane using an in-house stochastic version of TurboReg affine transformation (Thevenaz et al., 1998). The alignment step zero-pads the images in order to maintain a uniform size, which are subsequently cropped to yield the final 3D image volumes. The registration and edge cropping process yields a final resolution of 5,634 × 1,912 × 757 for Bx1, 5,728 × 3,511 × 2,526 for Bx2, 5,990 × 3,812 × 1,884 for Bx4, 6,065 × 3,976 × 792 for PTT, 6,114 × 3,874 × 1,583 for PDAC, and 6,083 × 3,740 × 2,208 for MCF7, as shown in Figure 1B. The third dimension refers to the number of slices in each stack. Intermittently, the brightness of a few images in the stack varied, increasing the complexity of the images and making segmentation more challenging. Histogram equalization was applied to ensure consistency across the stack and reduce complexity of the images.

The cells and organelles were manually labeled using Microscopy Image Browser (Belevich et al., 2016). The availability of ground truth labels over different datasets is shown in Table 1. Nuclei and nucleoli are labeled on all slices of PTT, Bx1, and Bx2 datasets and only 19, 16, and 24 slices of Bx4, PDAC, and MCF7 datasets, respectively. Mitochondria are labeled on all slices of Bx1 and Bx2 datasets and only 13 and 10 slices of PDAC and MCF7 datasets, respectively. Only the PDAC dataset has all cell membranes labeled on all slices. For Bx1 and Bx2 datasets, six and 11 cells are selected, respectively, and labeled completely across all the slices in the datasets. The neural network employed in Section 3.3 would encounter confusion if some cells in a training image were labeled as cells and others as background. To minimize potential confusion during the training process, we selected 10, 23, and 22 slices from the Bx1, Bx2, and MCF7 datasets, respectively, and labeled the cell membranes of all the cells present in these selected slices. Lysosomes and endosomes are labeled on 13 slices in the Bx2, 13 slices in the PDAC, and 14 slices in the MCF7 datasets, while only endosomes are labeled on 10 slices in the Bx1 dataset. Sparse labeling of nuclei and nucleoli takes 1–2 days, and that of mitochondria, lysosomes, endosomes, and cell membranes takes approximately 5–10 days each. Manual labeling of nuclei and nucleoli on all images of PTT, Bx1, and Bx2 datasets required approximately 50–80 h each. Manual labeling of mitochondria and cells on all images of the Bx2 dataset required approximately 8 months each.

**TABLE 1:** Availability of ground truth labels over different datasets. *✓✓*denotes all images labeled, *✓*denotes images sparsely labeled, and a blank denotes no images labeled.

Type	Dataset	Cells	Nuclei	Nucleoli	Mitochondria	Lysosomes	Endosomes
Breast cancer	Bx1	*✓✓*	*✓✓*	*✓✓*	*✓✓*		*✓*
	Bx2	*✓✓*	*✓✓*	*✓✓*	*✓✓*	*✓*	*✓*
	Bx4		*✓*	*✓*			
Pancreatic cancer	PTT		*✓✓*	*✓✓*			
	PDAC	*✓✓*	*✓*	*✓*	*✓*	*✓*	*✓*
3D-cultured breast cancer cells	MCF7	*✓*	*✓*	*✓*	*✓*	*✓*	*✓*

### 3.3 Proposed approach

We aim to segment the cell membranes and five cell organelles—nuclei, nucleoli, mitochondria, endosomes, and lysosomes—from 3D FIB-SEM images. As the organelles mostly appear as distant objects with boundaries, they can be segmented using a semantic segmentation network. We use ResUNet as the base network for semantic segmentation, where we combine residual blocks with the U-Net architecture. The proposed network consists of an encoding path that extracts features and a decoding path that upsamples the extracted features to obtain full-resolution segmentation but uses residual blocks of convolutional layers as building units (Figure 1D). A residual block consists of two convolutional layers with a kernel size of 3 × 3, each preceded by batch normalization and a rectified linear unit (ReLU), along with a residual shortcut connection (Figure 1C).

The encoding path in the proposed network consists of four residual blocks. We use a strided convolution in the first layer of the residual block instead of a pooling layer to downsample the feature maps. The number of feature maps is doubled along each successive block in the encoding path to enable richer feature extraction. The encoding path is followed by a residual block, which acts as a bridge between the encoding and decoding paths. Corresponding to the encoding path, the decoding path also consists of four residual blocks. The decoding path begins with the upsampling of the feature maps found in the previous level of the decoding path, followed by a 2 × 2 convolution to half the number of feature maps. These feature maps are then concatenated with the feature maps at the same level in the encoding path through a skip connection. This concatenation step helps combine the deep, semantic, coarse-grained feature maps from the decoder path with high-resolution feature maps from the encoder path, enabling effective recovery of fine-grained details. These concatenated feature maps are then passed through the residual block. The output of the last residual block is passed through a 1 × 1 convolutional layer, followed by sigmoid activation to provide the final segmentation mask. In Section 5.3, we compare the segmentation results of ResUNet with those of TransUNet, a hybrid CNN–Transformer architecture that leverages both spatial information from CNN features and global context from Transformers (Chen et al., 2021), and SETR, a pure Transformer-based encoder combined with a simple decoder that achieved state-of-the-art results on several large image segmentation datasets (Zheng et al., 2021). Leveraging the similarities of structures contained in a vEM image stack, the neural network is trained on a small subset of manually labeled 2D images evenly distributed in the vEM image stack to efficiently segment the rest of the images in the stack.

As discussed previously, the cell organelles are relatively well defined and have a clear boundary around them; therefore, they can be segmented with semantic segmentation networks like ResUNet. However, cancer cells in electron microscopy images do not always have clear boundaries separating them; therefore, ResUNet segments all cells together as one big blob. Furthermore, cancer cells have filopodium-like protrusions, which are thin, long, finger-like protrusions from the cell membrane that act like antennas to probe the surrounding environment. While capturing the FIB-SEM image, if the filopodium-like protrusions are cut perpendicular to their length, they appear as island-like blobs detached from the main cell (Supplementary Video S1). In order to capture the main body and the filopodium-like protrusions, we propose a multi-pronged approach combining segmentation, propagation, and tracking strategies for cell segmentation.

Instead of training ResUNet to segment only the cell interior region, we train the network to segment the cell boundaries, along with the cell interior region. To do so, we provide two ground truth maps to the network, one containing the cell masks and another containing the cell boundaries, from a small subset of manually labeled 2D images evenly distributed in the vEM image stack. The cell mask map is an image with all pixels representing the cell interior region marked 1. Thus, using ResUNet, we segment the cell interior, cell boundaries, and the protrusions that appear as island-like blobs. However, the boundary segmentation from the neural network alone could not separate adjacent cells with similar intensity and texture variations. In order to obtain a precise separation of adjacent cells with similar appearances, the boundary information from the neural network is combined with the boundary propagated using optical flow from manually labeled 2D images.

Optical flow provides the flow vectors representing the apparent motion of individual pixels between two images. We use the Farneback algorithm as it computes a dense optical flow—a flow vector for each pixel (Farnebäck, 2003). The Farneback algorithm generates an image pyramid to estimate displacement at multi-scales, starting at a coarser level and refining the estimate on finer levels. The pyramid decomposition enables the algorithm to handle both large and small pixel motions. The parameter settings of the algorithm that work best for our images are the number of pyramid levels = 6, neighborhood size = 5, filter size = 30, pyramid scale = 0.2, and number of iterations = 3.

We experiment three ways of combining boundary information from the optical flow and cell interior mask from ResUNet in separating the cells. First, we directly overlay the optical flow boundaries on the cell interior mask prediction from ResUNet. Second, we selectively include cell boundaries propagated using optical flow only in regions with overlapping cells. In the first two methods, the propagation of cell boundaries using optical flow was performed as a separate task independent of the estimate from the segmentation model. In order to obtain a better continuity of the cell boundary, instead of propagating the cell boundaries from the manually labeled image to all the following images, we propagate it only onto the image immediately following the manually labeled image. We then use the second method to combine it with the boundary from the ResUNet output of that image to obtain its cell segmentation. Optical flow is then applied to these newly estimated cell boundaries, which have the combined information from ResUNet and optical flow. This is the third method of using the boundary information to separate cells. Once we combine the boundary information using one of these methods, it is then overlaid on the cell-interior mask segmented using ResUNet. Individual cells are then separated from the cell-interior mask by performing watershed (a region-growing-based segmentation method) (Beucher, 1992; Barnes et al., 2014) using centroids of cells in the nearest manually labeled image as seeds.

With individual cells and the island-like blobs segmented in each image, the next step is to track each of these regions across images and associate the protrusions to their corresponding cells. We use an overlap-based label propagation technique to obtain the cell associations. Intersection over union (IoU) is a metric that measures the overlap between two regions. Here, the IoU metric given in Eq. 1 is used to track isolated regions across images.

$$\text{IoU}=\frac{\text{Area of overlap}}{\text{Area of union}}.$$ (1)

In the first image in the stack, all isolated regions are given unique tracking labels. For each of the isolated regions in the next image, IoU is calculated against all the labeled regions in the previous image. Each isolated region is then assigned the tracking label of the region in the previous image with which it shares the highest IoU value. If there is no overlap and a new region is detected, a new tracking label is assigned to that region, which can then be tracked in subsequent frames. The individual main cell bodies of the cells in EM images appear bigger than the island-like blobs. We first run the tracking algorithm on only the individual main cells by using the cell IDs in the manually labeled images as the tracking labels. Once the main cell body is tracked across all the images, we track the island-like blobs to these cells. As the blobs are tracked across images, when they meet the main cell body, their label is modified to that of the main big cell, and therefore, all the regions associated with that blob (now a protrusion from a cell) in the previous slices will also have their label modified to that of the main cell. When a protrusion breaks off from a cell, it still retains the label of that cell. As there could be hundreds of blobs in one image, it would be inefficient to compare each blob with every other blob in the previous image. As the movement of regions between two successive images is small, the corresponding region, if present in the next image, would be in the vicinity of the region in the previous image and not in other far-off regions of the image. Therefore, instead of searching for overlapping regions all across the 6,000 × 4,000 image, we look for a region matching an existing track label in its local neighborhood of 512 × 512.

Once all cells and organelles are segmented, they can be rendered to visualize them in 3D, and quantitative image features can be extracted for further analysis.

### 3.4 Implementational details

The proposed network is implemented using the Keras framework (Gulli and Pal, 2017), with TensorFlow (Abadi et al., 2016) as the backend. The network is optimized by adaptive moment estimation (Adam) with a 10^–4^ learning rate, exponential decay rates for moment estimates *β*1 = 0.9 and *β*2 = 0.999, and epsilon = 10^–7^. It is trained to minimize the Dice loss function for 5,000 weight updates. The experiments were performed on a single NVIDIA Tesla P100 GPU.

### 3.5 Inference methodology

As the size of an EM image is much larger than that of an image processed by the network (512 × 512), each image is parsed into multiple overlapping tiles. Each tile is passed through the network to predict a probability of belonging to the foreground class for every pixel. The resulting overlapping segmentation maps are stitched together by multiplying each map with a 2D tapered cosine window and adding the result to reduce the edge artifact at tile borders. In the absence of blending or averaging operations, the resulting overlapping segmentation maps often exhibit a step effect-like artifact along the borders of the tiles. Finally, the resulting segmentation map is thresholded at 0.5, assigning all pixels with a value greater than or equal to 0.5 to the foreground class.

## 4 Quantification and statistical analysis

### 4.1 Evaluation metrics

The segmentation mask predictions were evaluated using three metrics: Dice coefficient, recall, and precision. The Dice coefficient provides a measure of the overlap between the detected output and the ground truth. It ranges between 0 and 1, where 1 denotes a perfect overlap and 0 denotes no overlap. The Dice coefficient is defined as

$$Dice=\frac{2\sum_{i}^{N}p_{i}g_{i}}{\sum_{i}^{N}p_{i}^{2}+\sum_{i}^{N}g_{i}^{2}}=\frac{2TP}{2TP+FP+FN},$$ (2)

where N is the total number of pixels, p is the predicted segmentation, g is the ground truth, TP is true positives, FP is false positives, and FN is false negatives. Recall is the fraction of foreground pixels correctly predicted as foreground, and precision is the fraction of the predicted pixels that actually belong to the foreground. If P is the predicted output and G is the ground truth, recall and precision are represented as

$$Recall=\frac{\left|P\cap G\right|}{\left|G\right|},$$ (3)

$$Precision=\frac{\left|P\cap G\right|}{\left|P\right|}.$$ (4)

### 4.2 Morphological and texture features

The segmentation of organelles allows us to characterize biologically relevant features such as morphology and texture. Morphology refers to the structure of the organelle. The morphological measures are designed to capture the size and shape and include features such as solidity, sphericity, and circular variance. *Solidity* quantifies the concavities of a surface and is calculated as the ratio of the volume of the object to the volume of the convex hull (smallest encompassing convex polygon) of the object.

$$\text{Solidity}=\frac{\text{Volume}}{\text{Convex Hull Volume}}.$$ (5)

*Sphericity* is a measure of how close an object resembles a sphere. It is defined as the ratio of the surface area of a sphere with the same volume as the object to the surface area of the object.

$$\text{Sphericity}=\frac{\pi^{1/3}{\left(6V\right)}^{2/3}}{A},$$ (6)

where *V* is the volume of the object and *A* is the surface area of the object (Wadell, 1935). Its value is 1 for a perfect sphere and decreases as the shape varies from a sphere. *Circularity variance* provides a measure of the spread of radii across the volume. Here, a radius denotes the distance between a point on the contour and the centroid (geometric center) of the volume. The lower the value, the tighter the clustering about a single mean.

$$\text{Circularity variance }\left(O\right)=\frac{1}{|Surf\left(O\right)|\mu_{r}^{2}}\underset{p\in Surf\left(O\right)}{\sum }{\left(\|p-C\|-\mu_{r}\right)}^{2},$$ (7)

where *Surf* is the surface contour of the object *O*, *μ*_*r*_ is its mean radius, and *C* is its centroid.

In addition to the standard morphological features, we design two new features to capture the properties of nucleoli in cancer cells. The nucleolus in cancer cells is characterized by the formation of fenestrations and movement toward the nuclear membrane. Fenestrations refer to all the cavities inside the nucleolus. We design two measures—percentage of volume fenestrated and proximity of the nucleolus to the nuclear membrane—to capture the abovementioned properties. The percentage of the fenestrated volume in the nucleoli is calculated as the ratio of the difference of filled-in volume and net volume to the filled-in volume. The volume is filled in by performing a fill-hole operation, as shown in Figure 1E. This measure is different from solidity as it measures the volume of cavities within the nucleolus, while solidity measures concavities in the outer surface.

$$\text{Fenestrated volume percentage}=\frac{\text{Filled volume}-\text{Net volume}}{\text{Filled volume}}.$$ (8)

If *d*_*cc*_ is the distance between centroids of the nucleus and nucleolus and *d*_*s*_ is the distance between the centroid of the nucleolus and the nearest point on the surface of the object, the proximity of the nucleolus to the nuclear membrane measure is calculated as follows:

$$\text{Proximity to nuclear membrane}=\frac{d_{cc}}{d_{cc}+d_{s}}.$$ (9)

The distances *d*_*cc*_ and *d*_*s*_ are shown in Figure 1F. It takes a value of 0 when the nucleolus is at the center of the nucleus and increases as it moves toward the nuclear membrane.

The texture features are designed to capture the spatial distribution of intensity patterns and include features from gray-level co-occurrence matrix (GLCM) (Gallowy, 1975), size zone matrix (SZM) (Thibault et al., 2013a; Thibault et al., 2013b; Thibault and Shafran, 2016), and power spectrum (Thibault et al., 2017). The GLCM examines the spatial relationship between pixels and defines how frequently a combination of pixel intensities occurs for a given offset. A GLCM was constructed by averaging the matrices obtained over 26 different offsets (Thibault et al., 2017). Four Haralick’s features, namely, homogeneity, correlation, variance, and contrast, were computed from the GLCM.

$$\text{Homogeneity}=\overset{N_{g}}{\underset{i=1}{\sum }}\overset{N_{g}}{\underset{j=1}{\sum }}\frac{p\left(i,j\right)}{1+{\left(i-j\right)}^{2}},$$ (10)

$$\text{Correlation}=\frac{\overset{N_{g}}{\underset{i=1}{\sum }}\overset{N_{g}}{\underset{j=1}{\sum }}\left(i\cdot j\right)p\left(i,j\right)-\mu_{x}\mu_{y}}{\sigma_{x}\sigma_{y}},$$ (11)

$$\text{Variance}=\overset{N_{g}}{\underset{i=1}{\sum }}\overset{N_{g}}{\underset{j=1}{\sum }}{\left(i-j\right)}^{2}p\left(i,j\right),$$ (12)

$$\text{Contrast}=\overset{N_{g}-1}{\underset{k=0}{\sum }}k^{2}\overset{N_{g}}{\underset{i=1}{\sum }}\overset{N_{g}}{\underset{j=1}{\sum }}\delta_{|i-j|,k}p\left(i,j\right),$$ (13)

where *p* (*i*, *j*) is the (*i*,*j*)th element of the GLCM, *N* the number of gray levels, and *μ* and *σ* denote the mean and standard deviation, respectively. Homogeneity measures the smoothness of the gray-level distribution in the image. The homogeneity is 1 for a diagonal GLCM. The correlation measures the joint probability occurrence of certain pixel pairs and is high if the gray level of the pixel pairs is highly correlated. Variance provides a measure of dispersion of the gray-level distribution and is large if gray levels are spread out. Contrast measures the local variations in the GLCM. The contrast value is low for smooth, soft textures and high for heterogeneous textures.

A gray-level SZM provides a statistical representation of the clusters of gray levels in the image (Thibault et al., 2013a; Thibault et al., 2013b; Thibault and Shafran, 2016). Each element *P* (*i*, *j*) in an SZM is equal to the number of zones of size *j* with gray level *i*. Unlike GLCM, SZM is rotation-invariant. Three features, namely, zone percentage, small-zone high gray-level emphasis (SZHGE), and the centroid of zone sizes, are extracted from SZM. The zone percentage measures the coarseness of the texture by taking the ratio of the number of zones (*N*_*z*_) to the number of voxels (N) in the image.

$$\text{Zone precentage}=\frac{N_{z}}{N}.$$ (14)

The SZHGE measure highlights zone counts in the left quadrant of SZM, where small zone sizes and high gray levels are located. The feature is defined as

$$\text{SZHGE}=\frac{\overset{N_{g}}{\underset{i=1}{\sum }}\overset{N_{s}}{\underset{j=1}{\sum }}P\left(i,j\right)i^{2}}{N_{z}},$$ (15)

where *N*_*s*_ is the total number of zones, *N*_*g*_ is the number of discretized gray levels present in the image, *N*_*z*_ is the maximum zone size of any group of linked voxels, and *s*_*ij*_ is the number of zones with a discretized gray level *i* and size *j*. The centroid of zone sizes is calculated as the centroid of SZM.

The power spectrum is a gray-level rotation and intensity-invariant texture descriptor (Thibault et al., 2017). It describes the shape and size of structures in an image. It is calculated by iteratively opening and closing an image with morphological operators and recording the resulting areas. Morphological operators are a set of image-processing operators that process images based on shapes. They apply a structuring element to an input image, creating an output image of the same size. A structuring element is a matrix with the size and shape of the object that needs to be analyzed in the input image. For example, to find a line, the structuring element represents a line. If *n* is the size factor for a structuring element, then the family of morphological openings $\Gamma ={\left(\gamma_{n}\right)}_{n\geq 0}$ and morphological closings $\Phi ={\left(\phi_{n}\right)}_{n\geq 0}$ can help study all the object sizes present in an image. The analysis of an image *f* with respect to an operator, for example, Γ, involves evaluating each opening of size *n* with a measurement *∫γ*_*n*_(*f*). The power spectrum curve of an image *f* with respect to Γ and Φ is defined as follows:

$$PS_{n}\left(f\right)=\frac{1}{\int f}\left\{\begin{array}{c} \int \gamma_{n}\left(f\right)-\int \gamma_{n+1}\left(f\right)\;\text{for}\;n\geq 0\;\\ \int \phi_{n}\left(f\right)-\int \phi_{n-1}\left(f\right)\;\text{for}\;n<0\; \end{array}\right..$$ (16)

It defines a probability distribution, and the moments of this distribution are employed as signature patterns to characterize textures. A peak in the power spectrum at a given scale *n* indicates the presence of many image structures of this scale or size.

## 5 Results

We applied the proposed segmentation approach on the six datasets comprising tissues from patients with breast and pancreatic cancer and a microspheroid prepared using a breast cancer cell line. Overall, we performed quantitative evaluation of cell, nucleus, and nucleolus segmentation on three datasets and mitochondrion segmentation on two datasets. In few datasets, some organelles were labeled only in a limited number of images, which were all used for training the segmentation model. In such cases, we present only qualitative results. For each dataset, a segmentation model was trained using a small subset of manually labeled images, and the trained model was used to generate segmentation masks on the rest of the unlabeled images. We used convolution and attention-based models for semantic segmentation.

### 5.1 Training data

The full resolution of the EM images cannot be analyzed directly by the network due to the memory restrictions of our GPU hardware. Therefore, an image crop size of 512 × 512 pixels is chosen in order to fit a meaningfully large number of images in a batch. We use a batch size of five in all our experiments, meaning that five image tiles are used to estimate the error gradient at each iteration of the network weight update. Using multiple training examples in a batch for error gradient estimation helps make the training process more stable. We choose a batch size of five as it is the maximum number of tiles that could fit in the GPU memory in our case. The nuclei occupied 15%–35% of the pixels in each image, and a random selection of five 512 × 512 tiles per image ensured that there was enough representation of the nucleus in a batch. Mitochondria, endosomes, lysosomes, and cell bodies also displayed good representation in five tiles as they were well distributed in the image. However, nucleoli are smaller and sparser, occupying less than 1% of a full image. As a result, if sampled randomly, a large number of tiles would need to be considered in order to form a batch with enough nucleolus representation, which would exceed the GPU memory. To ensure that the model encounters sufficient representation of nucleoli during training, the batch size is still maintained at five, but the tiles are selected such that at least four out of the five randomly selected tiles contain no less than 100 pixels related to the nucleolus. Horizontal and vertical flips are randomly applied as data augmentation steps.

### 5.2 Deep learning models can accurately segment intracellular organelles

**FIGURE 2:**
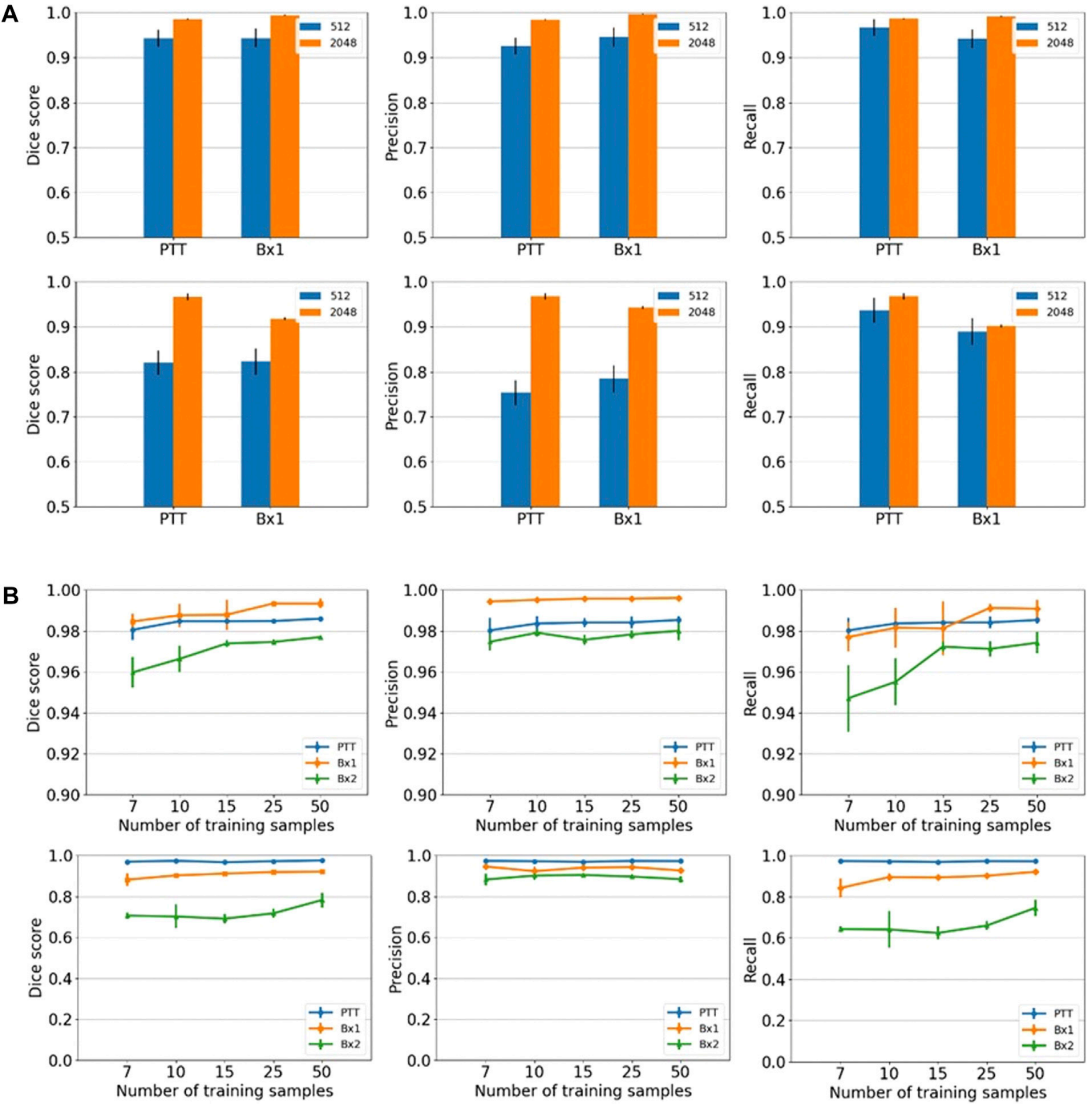
Nucleus and nucleolus segmentation performance. **(A)** Effect of image tile size (context window). Segmentation performances for nuclei (top row) and nucleoli (bottom row) using different input tile sizes measured by the Dice score (first column), precision (second column), and recall (third column) on the Bx1 and PTT datasets. The blue bar represents the results of training the network directly on the image tiles of size 512 × 512 pixels from the FIB-SEM images. The orange bar represents the results of training the network on the image tiles of size 2,048 × 2,048 pixels downsampled to 512 × 512 pixels, which provide larger contextual information. Each error bar represents 10 separate experiments in which a new model was trained from scratch using the specified number of training images. **(B)** Effect of training set size. Segmentation performances for nuclei (top row) and nucleoli (bottom row) using different training set sizes, measured by the Dice score (first column), precision (second column), and recall (third column) on PTT (blue), Bx1 (orange), and Bx2 (green) datasets. For each dataset, the performance was evaluated over training set sizes of 7, 10, 15, 25, and 50 in order to find the minimum number of images required to generate accurate segmentation. Each error bar represents the mean and standard deviation of results obtained from 10 separate experiments in which a new model was trained from scratch using the specified number of training images.

**FIGURE 3:**
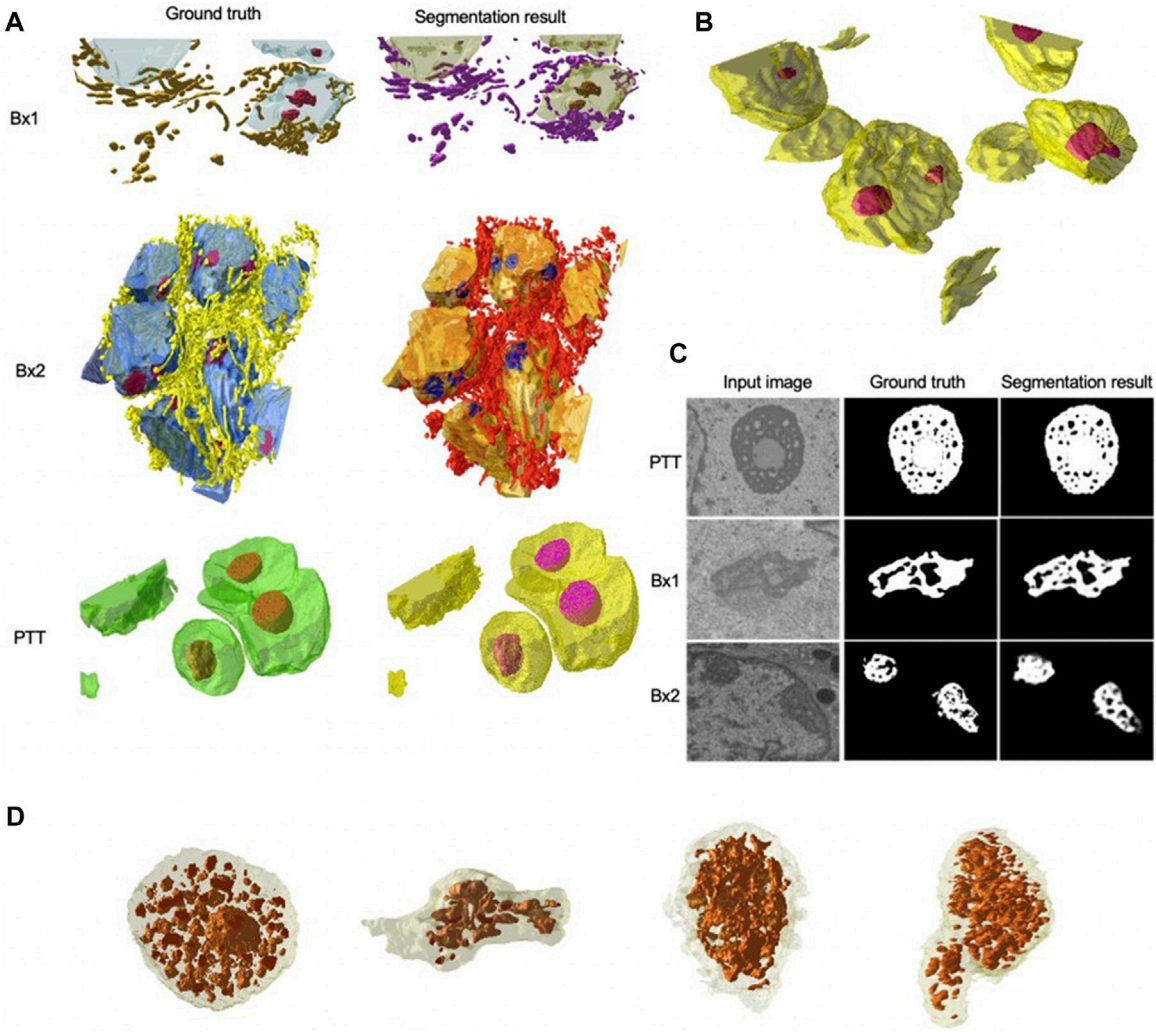
Nucleus and nucleolus volume renderings and nucleolus segmentation results. **(A)** Volume renderings showing the 3D structure of ground truth masks and predicted segmentation masks for PTT, Bx1, and Bx2 datasets. **(B)** Volume rendering showing the 3D structure of predicted segmentation masks for the Bx4 dataset. **(C)** Representative qualitative results showing input images (first column), ground truth (second column), and predicted nucleoli (third column) for PTT (first row), Bx1 (second row), and Bx2 (third row) datasets. **(D)** Volume renderings of the fenestrations in the nucleoli of PTT, Bx1, Bx2, and Bx4 datasets.

**FIGURE 4:**
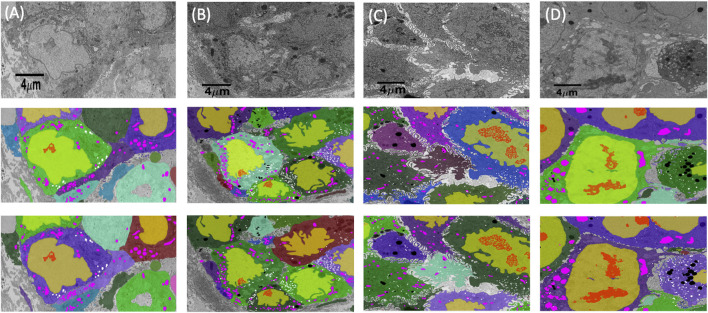
Cell and organelle segmentation results on Bx1, Bx2, PDAC, and MCF7 datasets. Qualitative results showing input images (first row) overlaid with nucleus (yellow), nucleolus (red), mitochondrion (pink), endosome (white), lysosome (black), and cell segmentation (random colors) on ground truth masks (second row) and predicted segmentation masks (third row) for **(A)** Bx1 **(B)** Bx2, **(C)** PDAC, and **(D)** MCF7 datasets.

**FIGURE 5:**
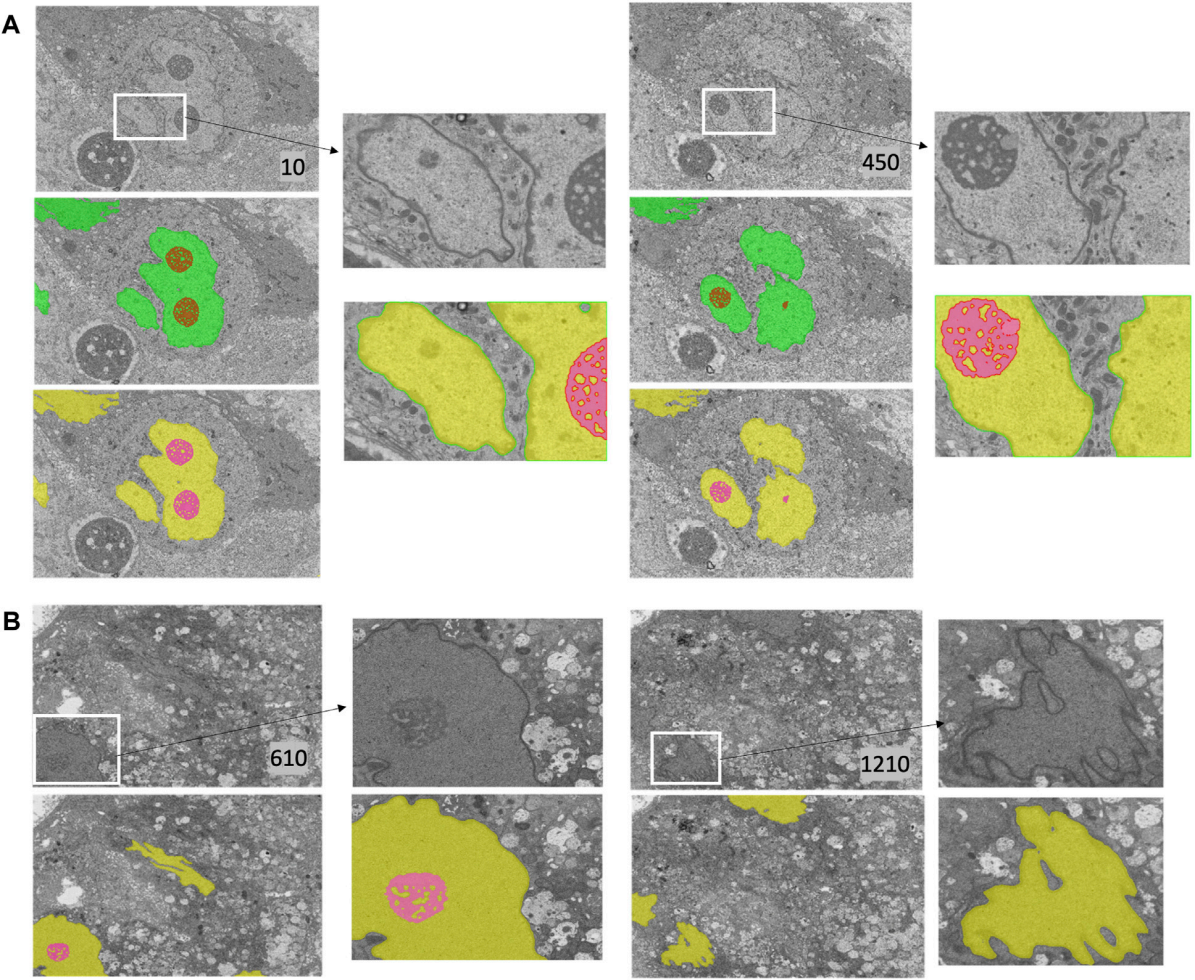
Nucleus and nucleolus segmentation results on PTT and Bx4 datasets. **(A)** Representative qualitative results showing input images (top row) overlaid with nucleus (green) and nucleolus (red) ground truth masks (middle row) and predicted nucleus (yellow) and nucleolus (pink) segmentation masks (bottom row) for the PTT dataset. **(B)** Representative qualitative results showing input images (top row) overlaid with predicted nucleus (yellow) and nucleolus (pink) segmentation masks (bottom row) for the Bx4 dataset. All 19 slices from the Bx4 dataset that had nucleolus labels were employed in the training of ResUNet, resulting in the possibility of conducting only a qualitative comparison. Numbers in the lower right-hand corner of images indicate the slice position of the image in the full image stack. Scale: horizontal image width = 25 *μm*.

We primarily used the convolution-based ResUNet as the segmentation model. To train the segmentation model, a sparse subset of training images (typically between 7 and 50 slices) was randomly selected from the 3D image stack and used to tune the network weights by minimizing the difference between the ground truth segmentation and the network-generated segmentation on these training slices. Once trained, the network was used to segment the rest of the slices in the stack that were not used for training, and performance in terms of the Dice score, precision, and recall was calculated. We randomly selected the input images 10 times, and the mean performance metrics over the 10 runs was reported. Figure 2B shows the performance metrics for nucleus and nucleolus segmentation in PTT, Bx1, and Bx2 datasets (nucleus results in the top row, and nucleolus results in the bottom row). Depending on the datasets and the training set size, overall Dice scores of 0.95–0.99 and 0.70–0.98 were achieved for nucleus and nucleolus segmentation, respectively. In addition to the nucleoli boundary, the model accurately segmented the fenestrations within the nucleoli (Figure 3C). Dice scores of 0.86 and 0.76 were obtained in segmenting mitochondria from Bx1 and Bx2 datasets, respectively. Segmentation performances of endosomes and lysosomes were evaluated qualitatively (Figure 4) as they are labeled only on a few slices that are used to train the segmentation model. Figure 3A displays the comparison between volumes rendered from manual annotations and those from predicted segmentation, with nucleoli nested inside the nuclei for PTT, Bx1, and Bx2 datasets. Figure 3B displays the volume rendered from the predicted segmentation for the Bx4 dataset as it does not have ground truth labels for the entire stack. Figure 3D displays the volume rendering of the fenestrations in the nucleoli of PTT, Bx1, Bx2, and Bx4 datasets. The 2D image slices overlaid with ground truth and predicted segmentation for PTT and Bx4 datasets are shown in Figure 5, and for Bx1, Bx2, PDAC, and MCF7 datasets, they are shown in Figure 4. They illustrate the accuracy of model segmentation compared to the ground truth annotation.

### 5.3 Larger context improves segmentation performances

The cancer cells in EM images are relatively large. A tile of size 512 × 512 pixels typically includes only a small section of the cell (for example, a small part of the nucleus) and therefore does not contain enough spatial context. We hypothesize that providing better contextual information regarding the surroundings of the organelles could lead to improved segmentation. In order to incorporate a more global context while subjected to GPU memory restrictions, we extracted tiles of size 2,048 × 2,048 pixels and downsampled them to 512 × 512 pixels and trained segmentation models on these downsampled tiles. We also conducted experiments using tiles of sizes 1,024 × 1,024 and 3,072 × 3,072 pixels and found that tiles of size 2,048 × 2,048 pixels produced superior segmentation results. We also compared the segmentation performance of fully convolutional ResUNet with the more recent TransUNet and SETR, where the global context is captured by transformers with a self-attention mechanism for the segmentation of nuclei.

**FIGURE 6:**
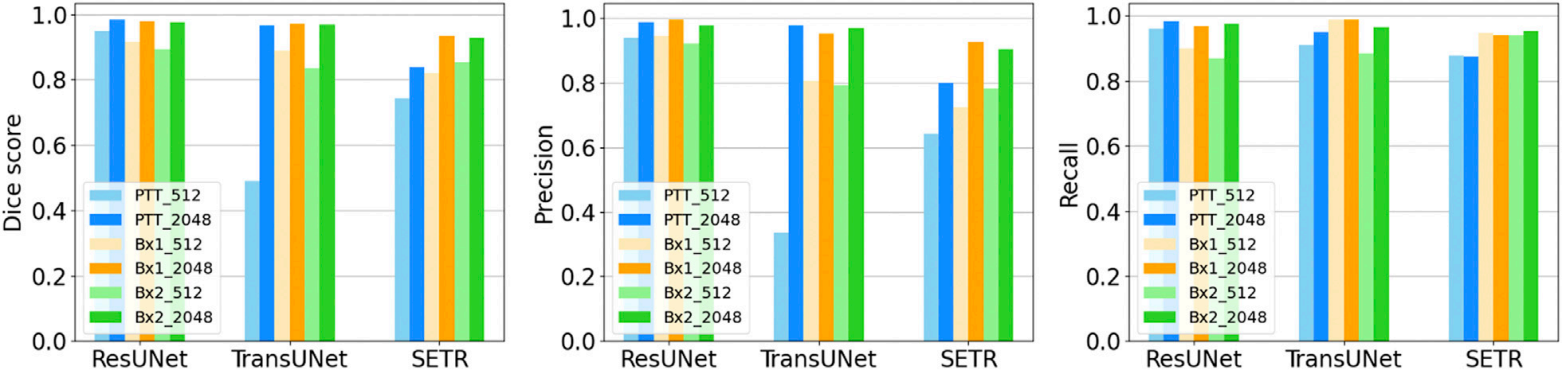
Nucleus segmentation performance using ResUNet, TransUNet, and SETR. Segmentation performances for nuclei using different input tile sizes measured by the Dice score (first column), precision (second column), and recall (third column) on the PTT, Bx1, and Bx2 datasets. The lighter bar represents the results of training the network directly on the tiles of size 512 × 512 pixels from the FIB-SEM images. The darker bar represents the results of training the network on image tiles of size 2,048 × 2,048 pixels downsampled to 512 × 512 pixels, which provide larger contextual information.

Providing a larger context during training improved all three metrics in nucleus (Figure 2A, top row) and nucleolus (Figure 2A, bottom row) segmentation. The information provided by the larger context seemed to outweigh any information loss due to downsampling. Furthermore, using a larger tile size enabled faster processing of an entire EM image, as fewer tiles were required to reconstruct the final result. The performance of TransUNet and SETR was on par or slightly lower than that of ResUNet and did not provide any added advantage in segmentation, as shown in Figure 6.

### 5.4 A small training set is adequate, and a larger training set brings further improvement

We varied the number of manually labeled full images used for training by selecting 7, 10, 15, 25, or 50 training images evenly distributed across the image stack. The models were trained on tiles of size 2,048 × 2,048 pixels downsampled to 512 × 512 pixels. The model performed well with only seven training images, with an overall Dice score of 0.95–0.99 for nuclei (Figure 2B, top row) and a Dice score of 0.70–0.98 for nucleoli (Figure 2B, bottom row), depending on the datasets. The performance continued to improve with more training images for all three metrics for both nuclei and nucleoli, reaching a Dice score in the range of 0.97–0.99 for nuclei and 0.80–0.99 for nucleoli with 50 training images. Even with 50 training images, it is still a very small training set, representing only 2%–5% of all the images in the stack. This illustrates that it is possible to train a reliable model with sparse manual labeling for vEM segmentation. The performances for the Bx2 dataset were lower than that for the other two datasets as it was a larger image stack exhibiting significant variability among the nuclei and nucleoli within the dataset.

### 5.5 Cell segmentation

**FIGURE 7:**
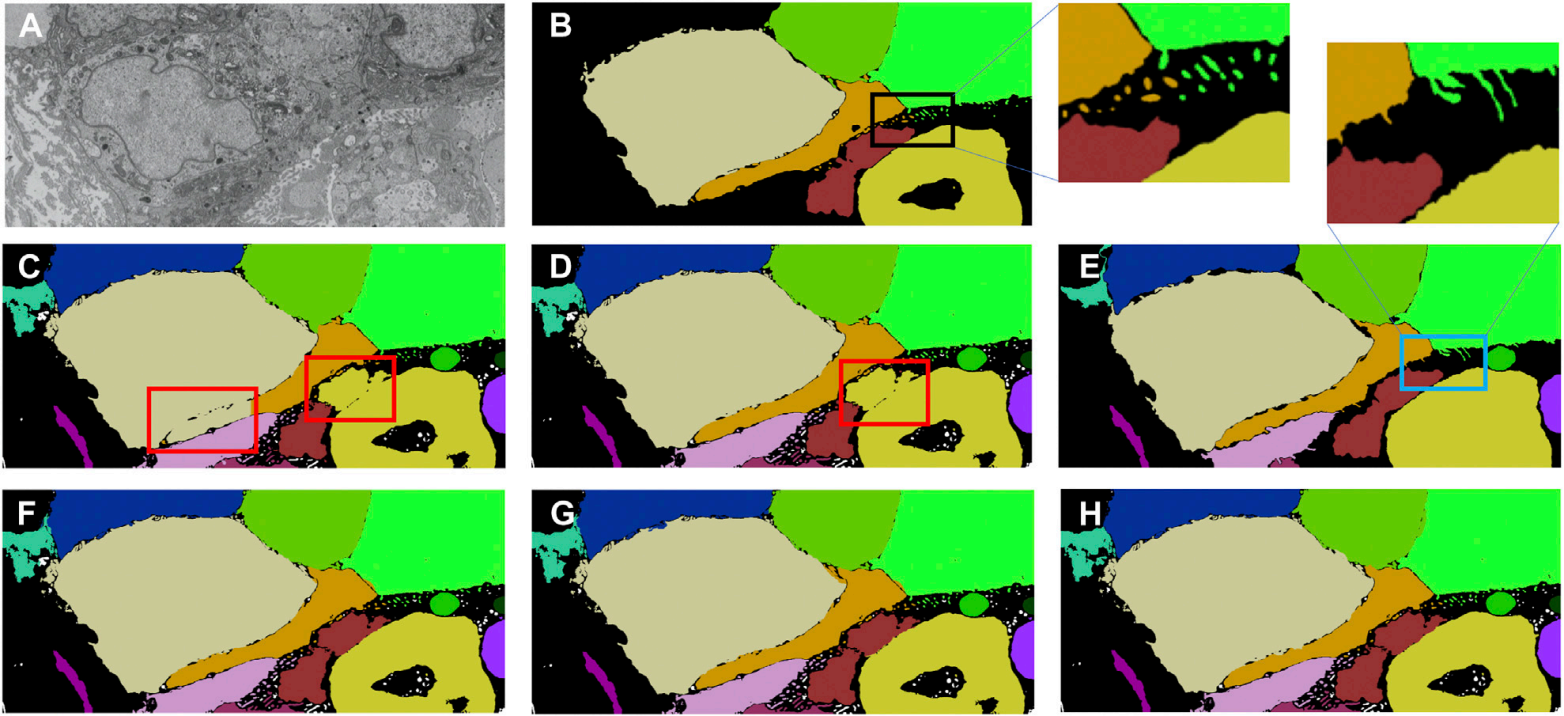
Cell segmentation results on the Bx1 dataset. Red boxes represent regions where the cells could not be separated accurately, and blue box represents regions where the cell protrusions are not segmented accurately. Representative qualitative results showing **(A)** input image, **(B)** ground truth segmentation (a few cells are not labeled in the ground truth), **(C)** segmentation of cells using a cell-interior mask alone, **(D)** segmentation of cells using a cell-interior mask and boundary predictions from the segmentation model, **(E)** segmentation of cells using optical flow alone, **(F)** segmentation of cells by overlaying boundaries propagated using optical flow on the cell-interior mask obtained from the segmentation model to separate cells, **(G)** segmentation of cells by selectively combining the optical flow boundary estimate with the boundary estimated from the segmentation model, and **(H)** segmentation of cells by propagating boundaries estimated in the previous frame using optical flow and combining with the boundary estimated from the segmentation model for the Bx1 dataset.

**FIGURE 8:**
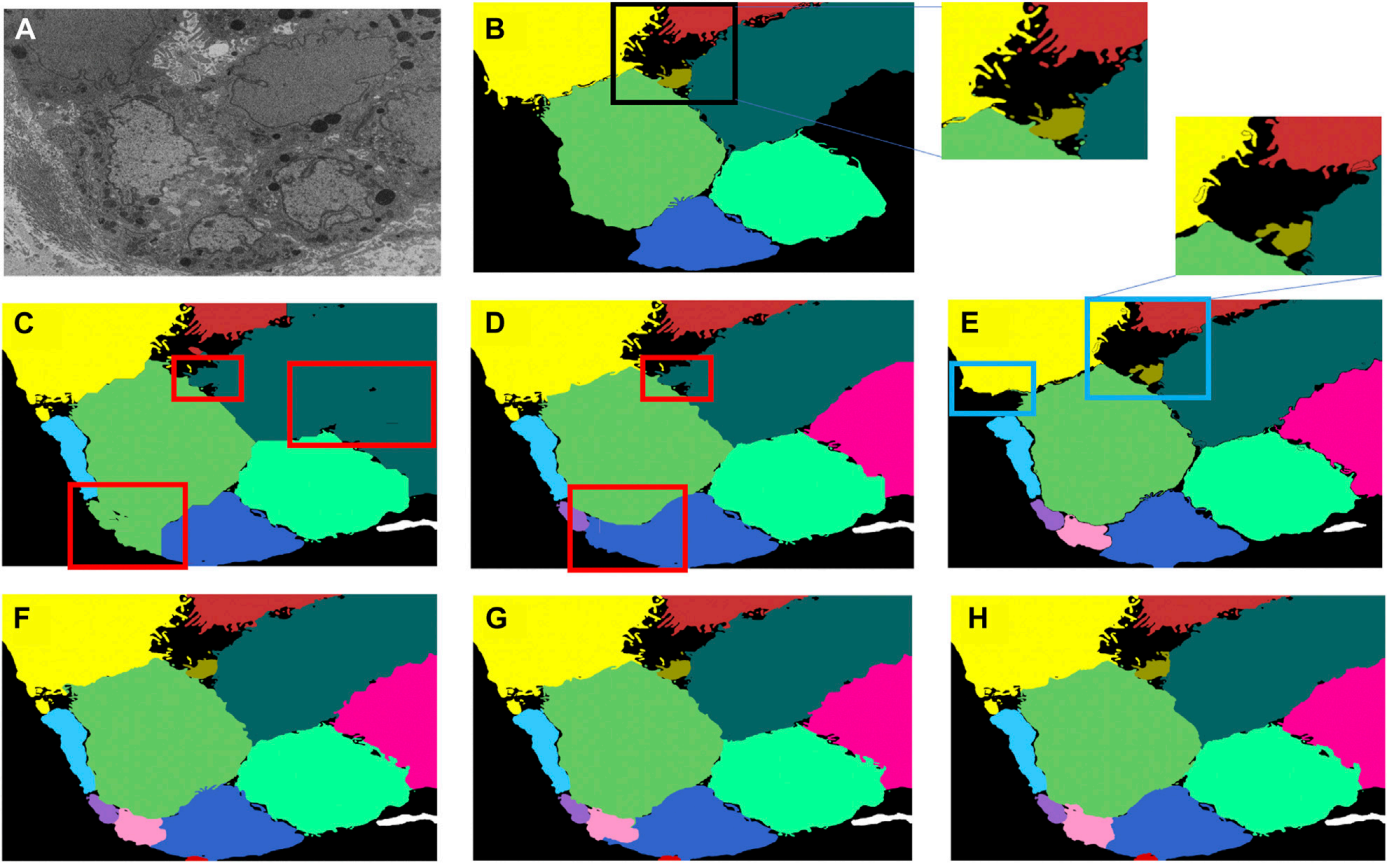
Cell segmentation results on the Bx2 dataset. Red boxes represent regions where the cells could not be separated accurately, and blue boxes represent regions where the cell protrusions are not segmented accurately. Representative qualitative results showing **(A)** input image, **(B)** ground truth segmentation (a few cells are not labeled in the ground truth), **(C)** segmentation of cells using a cell-interior mask alone, **(D)** segmentation of cells using a cell-interior mask and boundary predictions from the segmentation model, **(E)** segmentation of cells using optical flow alone, **(F)** segmentation of cells by overlaying boundaries propagated using optical flow on a cell-interior mask obtained from the segmentation model to separate cells, **(G)** segmentation of cells by selectively combining the optical flow boundary estimate with the boundary estimated from the segmentation model, and **(H)** segmentation of cells by propagating boundaries estimated in the previous frame using optical flow and combining with the boundary estimated from the segmentation model for the Bx2 dataset.

**FIGURE 9:**
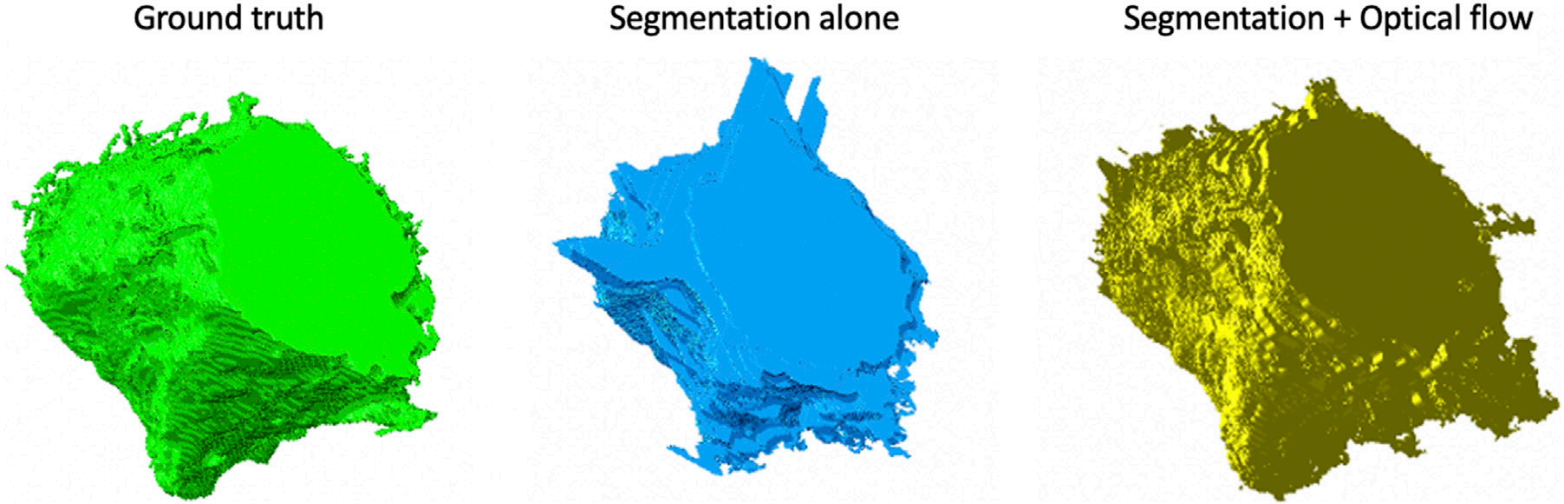
Volume rendering of a cell from the Bx2 dataset. Volume rendering of the (left to right) **(A)** ground truth, **(B)** cell segmented by using the watershed algorithm on the cell-interior mask from ResUNet, and **(C)** cell segmented by using the watershed algorithm on the cell-interior mask with boundary information from both ResUNet and optical flow.

A multi-pronged approach combining segmentation, propagation, and tracking strategies is used for segmenting cells, as described in Section 3.3. We trained ResUNet to segment the cell-interior mask and boundaries of all cells in the EM images. ResUNet provided precise cell-interior mask segmentation. However, the boundary segmentation from ResUNet could not separate adjacent cells that looked alike (Figure 7D; Figure 8D). Optical flow was used to propagate the cell boundaries across images. We observed that, as we moved farther from the ground truth image, the accuracy of the propagated boundary decreased. Furthermore, each filopodium-like protrusion of a cancer cell, resembling a separate island-like blob detached from the cell body, extends across only a few images within the stack. Therefore, optical flow cannot track such islands if they are not present in the ground truth image and also cannot detect the start and end of such islands (Figures 7E, 8E). We combined the strengths of ResUNet and optical flow by taking the boundary segmentation from ResUNet and adding to the boundary propagated by optical flow in regions where ResUNet could not predict the boundary (in the presence of similar adjacent cells). By doing so, we retained the precise boundary segmentation from ResUNet while benefiting from the cell separating ability of the optical flow estimate. As mentioned in Section 3.3, we used different approaches of combining the segmentation and optical flow boundary estimates. First, we directly overlaid the optical flow boundaries on the cell-interior mask prediction and separated cells using the watershed algorithm (Figures 7F, 8F). We observed that as optical flow does not move the boundary accurately, it resulted in a step effect in the final 3D reconstruction of segmentation. Therefore, we selectively include cell boundaries propagated using optical flow only in regions with overlapping cells. This helps retain the accurate cell boundaries from the segmentation model while separating overlapping cells (Figure 9; Figures 7G, 8G). In order to obtain a better continuity of the cell boundary, we propagate the combined cell boundary estimate of the previous image instead of the optical flow estimate alone (Figures 7H, 8H). All three methods of combining the segmentation and propagation boundary results improved the overall cell segmentation performance compared to using the segmentation or propagation method alone, as shown in Table 2 and Figure 9. In the PDAC dataset, the cells mostly had an extracellular matrix between them, and therefore, the cell-interior mask segmentation from ResUNet could result in good separation of cells, as shown in Table 2. However, in cases where filopodium-like protrusions came into contact with each other, the combined method with the propagation of the boundary using optical flow based on the boundary estimate of the previous image produced better separation of the filopodium-like protrusions.

**TABLE 2:** Cell segmentation performance measured using the Dice score in Bx1, Bx2, and PDAC datasets.

		Bx1	Bx2	PDAC
Segmentation alone	Mask	0.900	0.704	0.889
	Mask + border	0.927	0.841	0.888
Propagation alone	Optical flow	0.936	0.881	0.888
Segmentation + propagation	Mask + optical flow (OF)	0.952	0.902	0.888
	Mask + border + OF (selective)	0.950	0.900	0.888
	Mask + border + OF (propagate through frames)	0.952	0.894	0.888

**FIGURE 10:**
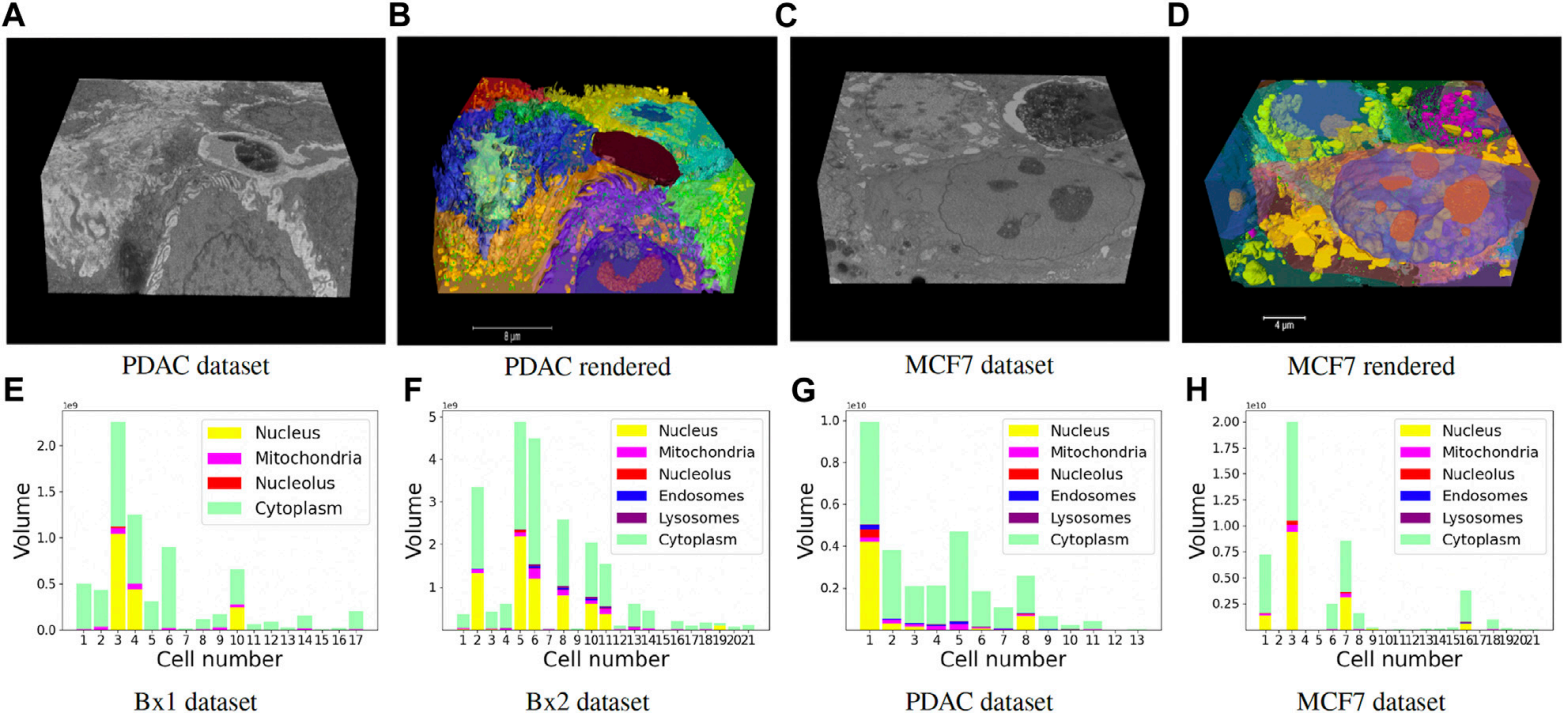
**(A–D)** Volume renderings showing the FIB-SEM volume and the predicted cell and organelle segmentations in PDAC and MCF7 datasets. **(E–H)** Volume occupied by the predicted organelles for each cell in each dataset.

Finally, we track the islands to the main cell bodies by calculating the intersection over union measure for all regions detected in consecutive images and associating regions with maximum overlap. As movement of regions between frames is minute, an overlap-based measure works well in tracking protrusions and cells in EM images. Volume renderings showing the FIB-SEM volume and the predicted cell and organelle segmentations for PDAC and MCF7 datasets are shown in Figures 10B, D and Supplementary Videos S2, S3. Segmentation of cells enables quantitative cellular analysis, where we can localize organelles to a cell and estimate their variations in density, size, and number among different cells. Figures 10E–H show the volume occupied by the predicted organelles for each cell in each dataset.

The training of the segmentation model took approximately 4 h, and prediction and splitting of the cells took approximately 2–3 h. The tracking of protrusions to main cells took a longer time as each image has to be processed serially, and the time also varied depending on the number of islands present in each image. It took approximately 1 min to process each image in the tracking of protrusion steps. Complete cell segmentation of Bx1, PDAC, Bx2, and MCF7 took approximately 1, 1.5, 2, and 2 days, respectively. This is a huge reduction where it takes months to manually segment cells in these datasets.

### 5.6 Quantitative characterization of nucleus and nucleolus morphology and texture

**FIGURE 11:**
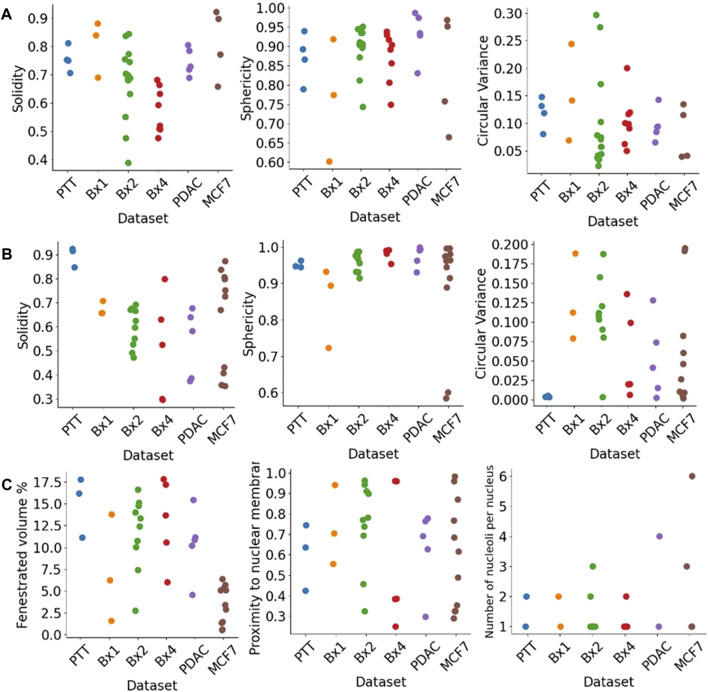
Morphological features extracted from nuclei and nucleoli. Solidity, sphericity, and circular variance measures for **(A)** nuclei and **(B)** nucleoli in PTT, Bx1, Bx2, and Bx4 datasets. **(C)** Percentage of the fenestrated volume in nucleoli and the proximity of nucleoli to the nuclear membrane for all datasets. Each dot represents the value of the feature for a nucleus or nucleolus.

The segmentation of nuclei and nucleoli allowed us to characterize biologically relevant features such as their morphology and texture. Figure 11 shows some of the morphological features we extract for nuclei (Figure 11A) and nucleoli (Figures 11B, C). The solidity feature, measuring the concavity of a surface, captures the nuclear envelope invaginations. The higher level of invaginations in Bx2 and Bx4 than in PTT, Bx1, PDAC, and MCF7 (Figure 3A) is reflected by their lower solidity value (Figure 11A). Similarly, the relatively smooth envelope of nucleoli in PTT is reflected by its higher solidity value (Figure 11B). The solidity of the nucleolus is calculated by filling the holes in the volume to exclude the effect of the volume fenestrated by pores and quantify only the overall change in shape. The sphericity and circular variance features measuring the roundness of an object captured shape irregularities in nuclei and nucleoli, which are common characteristics of cancer cells. Additionally, for the nucleolus, we calculated the percentage of volume fenestrated by pores. Its values were high for the PTT and Bx4 datasets as a result of the complex structure of pores within the nucleoli. Finally, varying levels of proximity of nucleoli to the nucleus membrane were also observed. This observation is consistent with published studies which suggest that nucleoli in cancer cells often move toward the nuclear membrane and form intranuclear canalicular systems between the nuclear membrane and nucleolus (Baba and Câtoi, 2007). Accordingly, the proximity of the nucleolus to the nuclear membrane feature captured the more centered positioning of nucleoli in the PTT dataset and the close proximity of nucleoli to the nuclear membrane in the Bx2 dataset. While most of the nuclei contained one nucleolus, several nuclei contained 2–6 nucleoli (Figure 11C).

**FIGURE 12:**
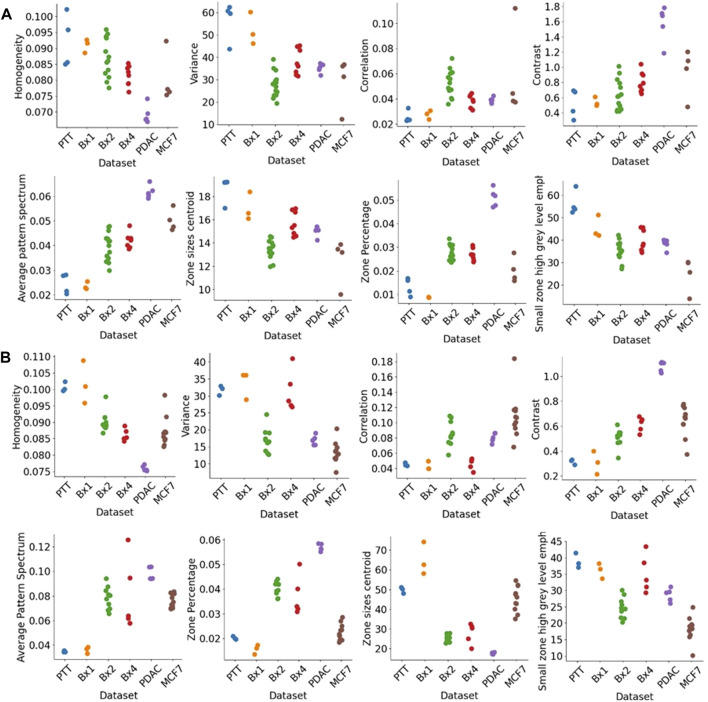
Texture features extracted from nuclei and nucleoli. GLCM features (top row), pattern spectrum, and SZM features (bottom row) for **(A)** nuclei and **(B)** nucleoli in PTT, Bx1, Bx2, and Bx4 datasets. Each dot represents the value of the feature for a nucleus or nucleolus.

Figure 12 shows the texture features extracted for nuclei (Figure 12A) and nucleoli (Figure 12B). We used three classic methods, GLCM, SZM, and pattern spectrum, to derive a set of texture features shown in Figure 12. These features capture the sizes of groups of voxels of similar gray-level intensities and can characterize the granularity of the chromatin structure in nuclei. These texture features can capture the differences between the tissue samples and can be potentially used for downstream analysis.

## 6 Discussion

We present here a framework for the segmentation of the cellular ultrastructure of cancer tissues from 3D FIB-SEM images, enabling rendering of the ultrastructure into interpretable forms and extraction of quantitative features. We used a ResUNet architecture to segment cells, nuclei, nucleoli, mitochondria, lysosomes, and endosomes, and evaluated the performance of the model on five human cancer biopsy samples acquired at OHSU and a microspheroid prepared using a breast cancer cell line. The ResUNet architecture was trained with different sizes of training datasets to evaluate the number of manually labeled images required for accurate segmentation. It was observed that the number of manually labeled images required greatly depended on the variability of the structure across the dataset. As the Bx2 dataset exhibited significant structural variations in nuclei and nucleoli compared to the Bx1 or PTT datasets, there was greater improvement in the Dice score with the increase in the number of images used during training. As there was less variability in Bx1 and PTT datasets, as few as seven labeled images were enough to train an efficient model. Even if 50 images had to be labeled, it constitutes a mere 2% of the Bx2 dataset and significantly reduces the labeling workload for the remaining 98% of the dataset. Furthermore, using image tiles of size 2,048 × 2,048 pixels downsampled to 512 × 512 pixels improved segmentation results compared to using images of 512 × 512 crops directly, as images with larger crops provided a greater context, which appeared to increase the segmentation performance. We demonstrated that ResUNet provided nucleus segmentation with Dice scores of 0.99, 0.99, and 0.98 and nucleolus segmentation with Dice scores of 0.98, 0.93, and 0.80 for PTT, Bx1, and Bx2 datasets and mitochondrion segmentation with Dice scores of 0.86 and 0.76 for Bx1 and Bx2 datasets, respectively. We presented a multi-pronged approach combining segmentation, propagation, and tracking strategies to segment cells in EM images. Combining propagation and segmentation methods helped accurately segment cells even in the presence of adjacent cells with similar intensities and texture variations. In the PDAC dataset, the cells mostly had an extracellular matrix between them, and therefore, using segmentation alone could result in good separation of cells. However, in cases where filopodium-like protrusions came into contact with each other, the combined method with the propagation of the boundary using optical flow based on the boundary estimate of the previous image produced better separation of the filopodium-like protrusions. The two extreme cases are when all cells in the dataset are either separate or closely packed, touching each other. When all cells are separate with an extracellular matrix between them, a segmentation method alone can result in good cell segmentation; when all cells are closely packed and adjacent cells look alike, only propagation-based methods can separate them. However, in most of the cancer datasets, only parts of the cells lie adjacent to another cell, and therefore, an approach combining the merits of segmentation and propagation methods is beneficial. The filopodium-like protrusions in cancer cells are an important feature in understanding the interactions of cancer cells, and therefore, it is necessary to segment them accurately. We demonstrated a tracking mechanism that can track the ends of filopodium-like protrusions that appear as island-like blobs detached from the main cell in few images and correctly associate them with their respective main cells.

Structures in 3D data collected via FIB-SEM exhibit high variability due to several factors, including the sample quality, tissue type, sample preparation techniques, microscope settings, and imaging pixel resolution (Navlakha et al., 2013). A small subset of the whole dataset contains enough information to capture most of the variability of a given structure with respect to the dataset, and we demonstrated that ResUNet trained with sparse labels generated segmentation masks for the rest of the images in the stack. Due to intensity variations caused by acquisition parameters and the variability of features among datasets, the generalizability of the segmentation models is currently limited. In order to have an automatic segmentation framework which does not necessitate sparse manual labeling for every new dataset, it is necessary to have a model that has enough representative training data. Our initial trials to segment individual 3D volumes by sparsely labeling each volume is a step toward building a dataset large enough to capture the variability among different organelles and the variability caused by different image acquisition settings.

Segmentation of cells and cellular ultrastructures allowed us to extract quantitative features from the datasets. We also demonstrate the feasibility of morphology and texture quantification in nuclei and nucleoli. These quantitative features can be extracted efficiently, robustly, and reproducibly. While it is beyond the scope of our work, we anticipate linking them to clinically relevant variables such as patient drug response in the future. This method can be extended to other cellular structures, enabling deeper analysis of inter- and intracellular state and interactions. The proposed segmentation of EM images fills the gap that limited the application of modern EM imaging in research and clinical practices. It will enable interpretative rendering and provide quantitative image features to be associated with the observed therapeutic responses.

## Data Availability

The raw data supporting the conclusion of this article will be made available by the authors, without undue reservation.
